# A prospective study on using shear wave elastography to predict the ypT0 stage of rectal cancer after neoadjuvant therapy: a new support for the watch-and-wait approach?

**DOI:** 10.3389/fmolb.2024.1402498

**Published:** 2024-04-26

**Authors:** Mengjia Liu, Ningyi Cui, Chao Sun, Xuantong Gong, Bo Wang, Di Yang, Yong Wang

**Affiliations:** Department of Ultrasound, National Cancer Center, National Clinical Research Center for Cancer, Cancer Hospital, Chinese Academy of Medical Sciences and Peking Union Medical College, Beijing, China

**Keywords:** rectal cancer, endorectal ultrasound, shear wave elastography, neoadjuvant chemoradiotherapy, pathological complete response

## Abstract

**Introduction:**

The diagnostic accuracy of traditional imaging examination in predicting ypT stage of rectal cancer after neoadjuvant therapy is significantly reduced, which would affect patients’ subsequent treatment choices. This study aimed to investigate the use of endorectal shear wave elastography (SWE) for diagnosing ypT0 stage of rectal cancer after neoadjuvant chemoradiotherapy (nCRT).

**Methods:**

Sixty patients with rectal cancer were prospectively recruited in this study. Data on endorectal ultrasound (ERUS) and SWE parameters were collected before nCRT and 6–8 weeks after nCRT. Postoperative pathological results were the gold standard for evaluating the diagnostic accuracy of SWE and ERUS in predicting the ypT0 stage of rectal cancer after nCRT. Receiver operating characteristic (ROC) curve analysis was used to determine the cut-off values of the SWE parameters that best corresponded to the ypT0 stage and analyze the sensitivity, specificity, and accuracy.

**Results:**

The diagnostic accuracies of using ERUS to predict the ypT and ypT0 stages of rectal cancer after nCRT were 58.1% (18/31) and 64.3% (9/14), respectively. The ROC curve was constructed with the lesion’s Emean, Emean corrected (EC), Emean difference (ED), Emean corrected differencede (ECD), Emean descendding rate (EDR) and Emean corrected descendding rate (ECDR) values after nCRT, the cut-off values of diagnosing the ypT0 stage were 64.40 kPa, 55.45 kPa, 72.55 kPa, 73.75 kPa, 50.15%, and 55.93%, respectively; the area under the curve (AUC) for diagnosing the ypT0 stage was 0.924, 0.933, 0.748, 0.729, 0.857 and 0.861, respectively. The EC value showed the best diagnostic performance.

**Conclusion:**

SWE could improve the accuracy of conventional ERUS in diagnosing the ypT0 stage of rectal cancer after nCRT. It is expected to become a new method to help predict pathological complete responses in clinical practice and provide new evidence for the watch-and-wait approach.

## Introduction

Rectal cancer is significant among newly occurring cancers worldwide, based on the latest report by the Global Cancer Observatory (Sung et al., 2021). The National Comprehensive Cancer Network guidelines (Benson et al., 2022) recommend neoadjuvant chemoradiotherapy (nCRT) combined with total mesorectal excision (TME) as the gold standard in patients with locally advanced rectal cancer (stage T3-4b). This is also recommended in patients with stage T2 low-position rectal cancer with a strong desire for anal preservation. However, the watch-and-wait (W&W) approach is a better choice in patients with clinical complete remission (cCR) after nCRT (Glynne-Jones et al., 2018). With the improvement in individualized treatment and recent development and application of new drugs, most patients experience tumor regression and downstaging after nCRT. Approximately 13.5%–40% (Fernández-Martos et al., 2010; Nilsson et al., 2013; Garcia-Aguilar et al., 2015; Perez et al., 2017; Zhang et al., 2020) can achieve pathological complete response (pCR). Systematic reviews (Li et al., 2016; Dossa et al., 2017) have found no significant differences regarding non-regrowth recurrence, cancer-specific mortality, disease-free survival, distant metastasis rates, and overall survival between patients managed with W&W and those with cCR undergoing surgery. Therefore, some patients may have been overtreated. Accurate diagnosis of ypT0 stage is the main aspect of confirming cCR. Conventional methods for ypT0 stage diagnosis after nCRT include magnetic resonance imaging (MRI), endoscopy, and digital rectal examination (MAAS et al., 2011). In patients with ypT0 stage, MRI shows substantial downsizing without residual tumor or residual fibrosis (with low signal on high b-value diffusion-weighted imaging [DWI]). Endoscopy shows no residual tumor or only a small residual erythematous ulcer or scar. Moreover, analysis of biopsies from scar, ulcer, or former tumor location reveals negative results. No palpable tumor is detected, when initially palpated with digital rectal examination. However, the current criteria cannot accurately guide for predicting ypT0 stage. Systematic reviews have shown that among patients diagnosed with cCR, the proportion of those achieving pCR was only 30% (Glynne-Jones et al., 2008).

The guidelines (Glynne-Jones et al., 2018; Diagnosis & Treatment Guidelines For Colorectal Cancer Working Group, 2019; Benson et al., 2022) recommend endorectal ultrasound (ERUS) as one of rectal cancer’s main imaging evaluation methods. A previous study (Wang et al., 2012) has shown satisfactory accuracy in diagnosing the T-staging of preoperative rectal cancer without neoadjuvant therapy using ERUS based on a technique that involves a sterile coupling gel filling the rectum, particularly in patients with early cancer (Tis and T1). However, pathological changes such as inflammation, edema, fibrosis, and necrosis occur in tumors and surrounding tissues and significantly decrease the accuracy of T staging in cancers after nCRT using ERUS (Dickman et al., 2013; Zhao et al., 2014; Tang et al., 2024).

Shear wave elastography (SWE) is a new elastic imaging technology that reflects the biomechanical characteristics of tissues in real-time and quantitatively measures lesion stiffness. SWE can provide information on stiffness changes in lesions before and after chemoradiotherapy to evaluate its efficacy, make up for the deficiency in conventional ERUS for differentiating inflammation from tumor, and improve the accuracy of ERUS in predicting ypT0. SWE is easy to perform, by which the results are more objective and reproducible compared with traditional strain elastography, which requires manual pressure. The application of SWE to patients with rectal cancer is in the preliminary research stage, and current studies mainly focus on the stiffness difference between benign and malignant rectal tumors, the initial stiffness value of tumors in different T stages, or the stiffness difference judgment of different T stages during downstaging (Fan et al., 2019; Cong et al., 2021; Loft et al., 2022a; Dong et al., 2023; Tang et al., 2024). There are few relevant studies on diagnosing the ypT0 stage using SWE after nCRT. Based on our previous study, the current study analyzed changes in stiffness before and after neoadjuvant therapy using SWE parameters and aimed to explore the application value of diagnosing the ypT0 stage and improving the diagnostic accuracy of ERUS in diagnosing rectal cancer after nCRT and provide more evidence for diagnosing patients with cCR in clinical practice.

## Materials and methods

### Patients

This study prospectively recruited patients with rectal cancer treated in our hospital between January 2023 and January 2024.

**FIGURE 1:**
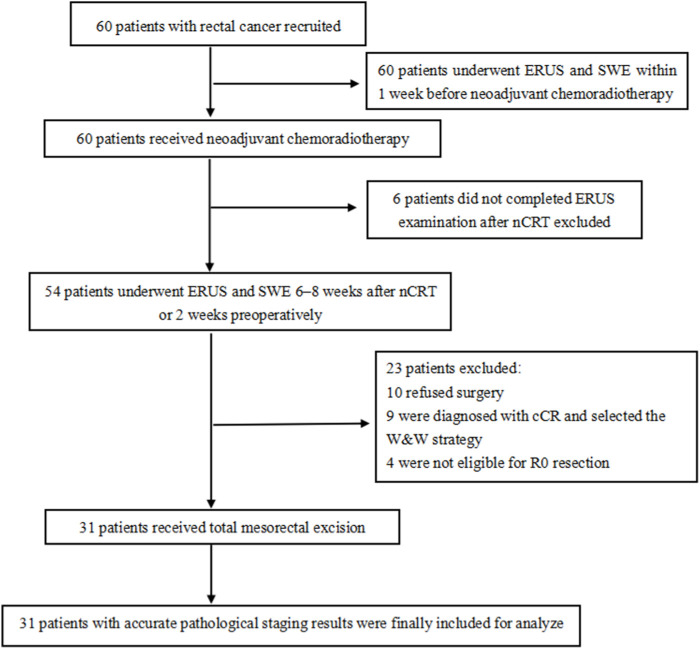
Flow chart of patients’ inclusion and exclusion. SWE: shear wave elastography, ERUS: endorectal ultrasound, nCRT: neoadjuvant chemoradiotherapy, cCR: clinical complete remission, W&W: watch-and-wait.

The inclusion criteria were:(1) rectal cancer confirmed by colonoscopic biopsy, (2) lower tumor margin <15 cm from the anal margin, and (3) consent to receive nCRT and TME treatment. The exclusion criteria were: (1) not undergoing TME surgical treatment; (2) tumor bleeding, obstruction, or intestinal stenosis and not completing ERUS examination; and (3) not undergoing timely examinations. Notably, all patients underwent ERUS and SWE within 1 week before nCRT initiation, 6–8 weeks after nCRT or 2 weeks preoperatively (Figure 1). The hospital Ethics Committee approved the ERUS and SWE examinations. Furthermore, two experienced sonographers jointly performed the ERUS and SWE assessments.

### Instruments and methods

All ERUS and SWE examinations were performed using a GE LOGIQ E11 (GE Healthcare,WI, USA) diagnostic apparatus equipped with an IC5-9-D end-fire type endorectal probe at a frequency of 5–9 MHz.

### ERUS examination

Patients underwent enemas to remove all air or stool from the rectum before the ERUS examination. Thereafter, the patients remained in the left lateral decubitus position, and approximately 100–150 mL coupling gel was injected directly into the rectum to ensure that the five layers of the intestinal wall and lesion could be clearly seen.

The tumors were evaluated for their location, length, thickness, echo pattern, color Doppler flow imaging, and the depth of the rectal wall layer invasion disrupted by the tumor. Ultrasound staging of the depth of rectal tumor invasion (uT) was performed using the Beynon staging criteria (Beynon et al., 1986): uT1 is a tumor confined to the mucosa or submucosa; uT2 is a tumor that breaks through the submucosa and invades the hypoechoic muscularis propria, whereas the adventitia is hyperechoic intact; uT3 is a tumor that breaks through the muscularis propria and reaches the serosa; and UT4 is a tumor that breaks through the complete layer and invades adjacent tissues or organs. The dynamic and static images of the scanned lesions were stored on a workstation.

### SWE examination

**FIGURE 2:**
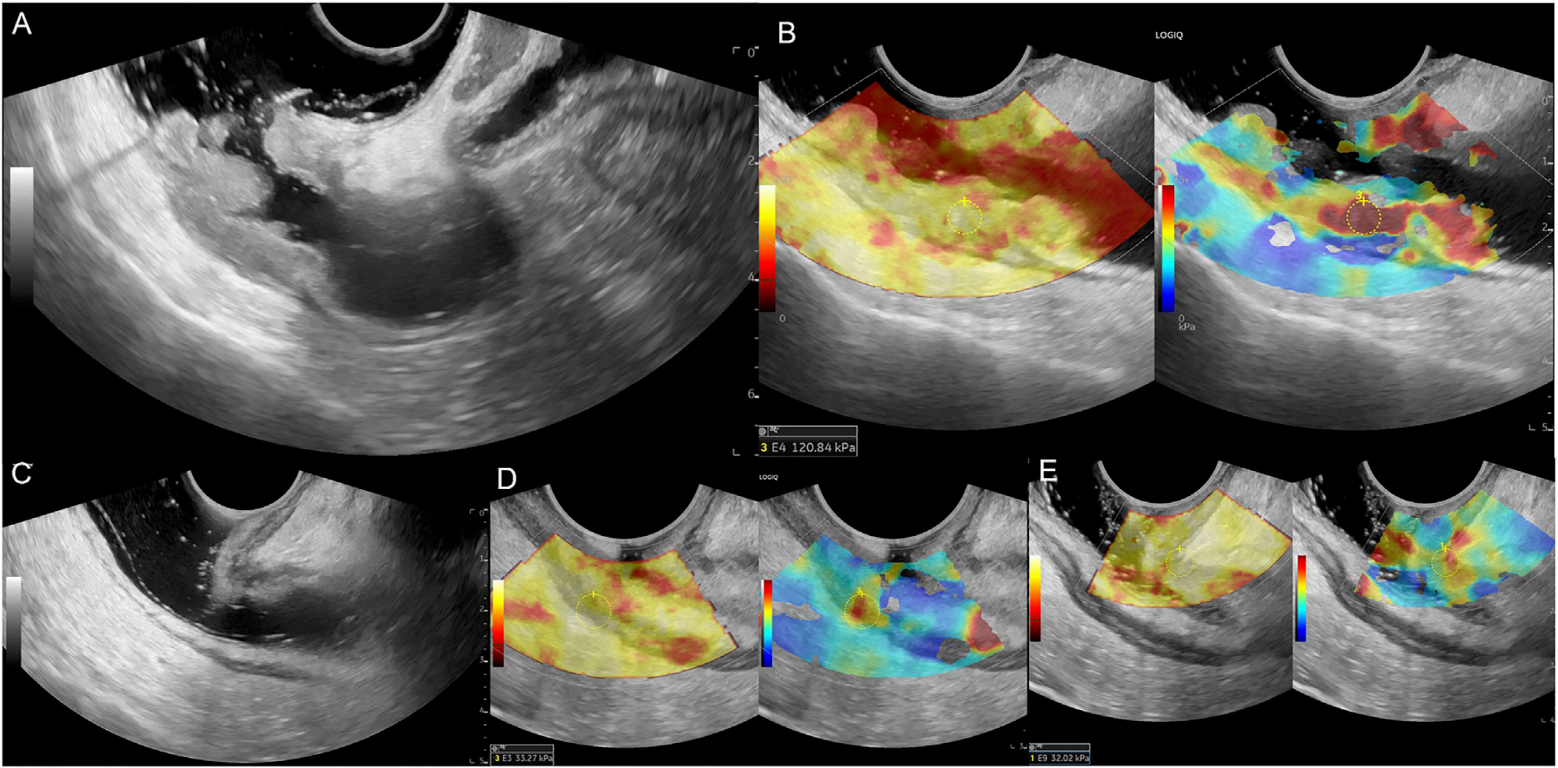
Male patient, 69 years old, **(A)** Middle and upper rectum circumferential protuberant lesion, gray-scale shown disruption of the rectal mucosa, submucosa, muscularis and serosa, ERUS staged uT3; **(B)** SWE showed the lesion is hard, pre-nCRT Emean = 120.84 kPa, pre-nCRT EC = 107.84kPa; **(C)** after nCRT, the wall of the middle and upper rectum was slightly thickened, especially the area of second rectal fold of the anterior wall, but the intestinal wall is clearly layered, ERUS staged uT0; **(D, E)** SWE showed the lesion area turn to be soft, choose the higher stiffness of the posterior rectal wall for calculating, post-nCRT Emean = 33.27kPa, post-nCRT EC = 26.27kPa, ED = 87.57kPa, ECD = 81.57kPa, EDR = 72.47%, ECDR = 75.64%, pathological result is ypT0N0.

After the ERUS examination, we switched to the elastic mode with a dual display of grayscale and elastography for the SWE examination. As shown in Figure 2B, the figure on leftside is the quality control image of gray-scale ultrasound, while the rightside image is the SWE image. A clearly displayed tumor section was selected, the probe was fixed, and the elastic sampling frame was adjusted to an appropriate range. The images were frozen after automatic scanning, the hardest part of the lesion and the normal intestinal wall were selected as the region of interest (ROI). Additionally, the mean Young’s modulus value (Emean, kPa) was measured using the Q-box, a quantitative measurement tool of the ultrasound instrument. Color coding of grayscale images reflects the quality control of elastography; yellow indicates a higher measurement quality, and red indicates a lower measurement quality. Color coding on the SWE images reflects tissue stiffness: blue represents low stiffness and low Emean values, and red represents high stiffness and high Emean values. The Q-box was round, with a diameter of 5 mm. The hardest area of the lesion was selected for measurement, which was repeated thrice; the average value was the lesion’s final Emean value. The intestinal wall above 1 cm from the edge of the tumor was selected as a normal wall and measured thrice; the average value was the intestinal wall’s Emean (EW, kPa).

The Emean value is the absolute value of the lesion measured using SWE. Owing to the differences in the normal intestinal wall stiffness values before and after nCRT in different patients, we calculated the difference as the Emean corrected value (EC, kPa), the Emean value subtracted from the EW value, to exclude the potential interference factors of the bowel wall.

The relative values of lesion stiffness changes before and after NCRT were also calculated. Emean difference (ED, kPa) was calculated by subtracting the post-nCRT Emean from the pre-nCRT Emean. Emean corrected difference (ECD, kPa) was calculated by subtracting post-nCRT EC from pre-nCRT EC. Emean descending rate (EDR, %) was defined as ED/pre-nCRT Emean × 100%. Emean corrected descending rate (ECDR, %) was defined as ECD/pre-nCRT EC × 100%.

### Statistical analysis

Statistical analysis was performed using SPSS 25.0 software, and the measurement data are expressed as mean ± standard deviation. A t-test was used to compare the quantitative data between two groups, and a one-way analysis of variance was used to compare quantitative data between multiple groups. The non-parametric rank-sum and chi-square tests were used to analyze data that did not conform to the normal distribution. Receiver operating characteristic (ROC) curves were used to evaluate the diagnostic efficacy of Emean, EC, ED, ECD, EDR, and ECDR in diagnosing the ypT stage of rectal cancer. Statistical significance was set at *p* ≤ 0.05.

## Results

### Identification initiative

This study prospectively enrolled 60 patients with rectal cancer who received nCRT. The patients aged 21–74 years (mean, 57.5 ± 11.8 years), with 45 male and 15 female individuals. There were 55 cases of T3 stage, two of T2 stage, and three of T4 stage before nCRT staging using diagnostic imaging.

Thirty-one patients who underwent TME and had accurate pathological staging results were included. They all had rectal adenocarcinoma. Of the other 29 patients, six patients did not complete the preoperative ERUS examination, 10 patients opted for conservative treatment and refused surgery, nine patients were diagnosed with cCR and selected the W&W strategy, and four patients were not eligible for R0 resection.

Of the 31 patients with surgical pathology, 23 (74.19%, 23/31) had downstaging, and eight (25.81%, 8/31) did not. Fourteen patients (45.16%, 14/31) yielded pCR of the ypT0 stage, whereas 17 (54.84%, 17/31) had a non-yield-pathological complete response (nypCR), including one (3.22%, 1/31) of the ypT1 stage, seven (22.58%, 7/31) of the ypT2 stage, eight (48.40%, 8/31) of the ypT3 stage, and one (3.22%, 1/31) of the ypT4 stage.

### ERUS evaluates T stage

**FIGURE 3:**
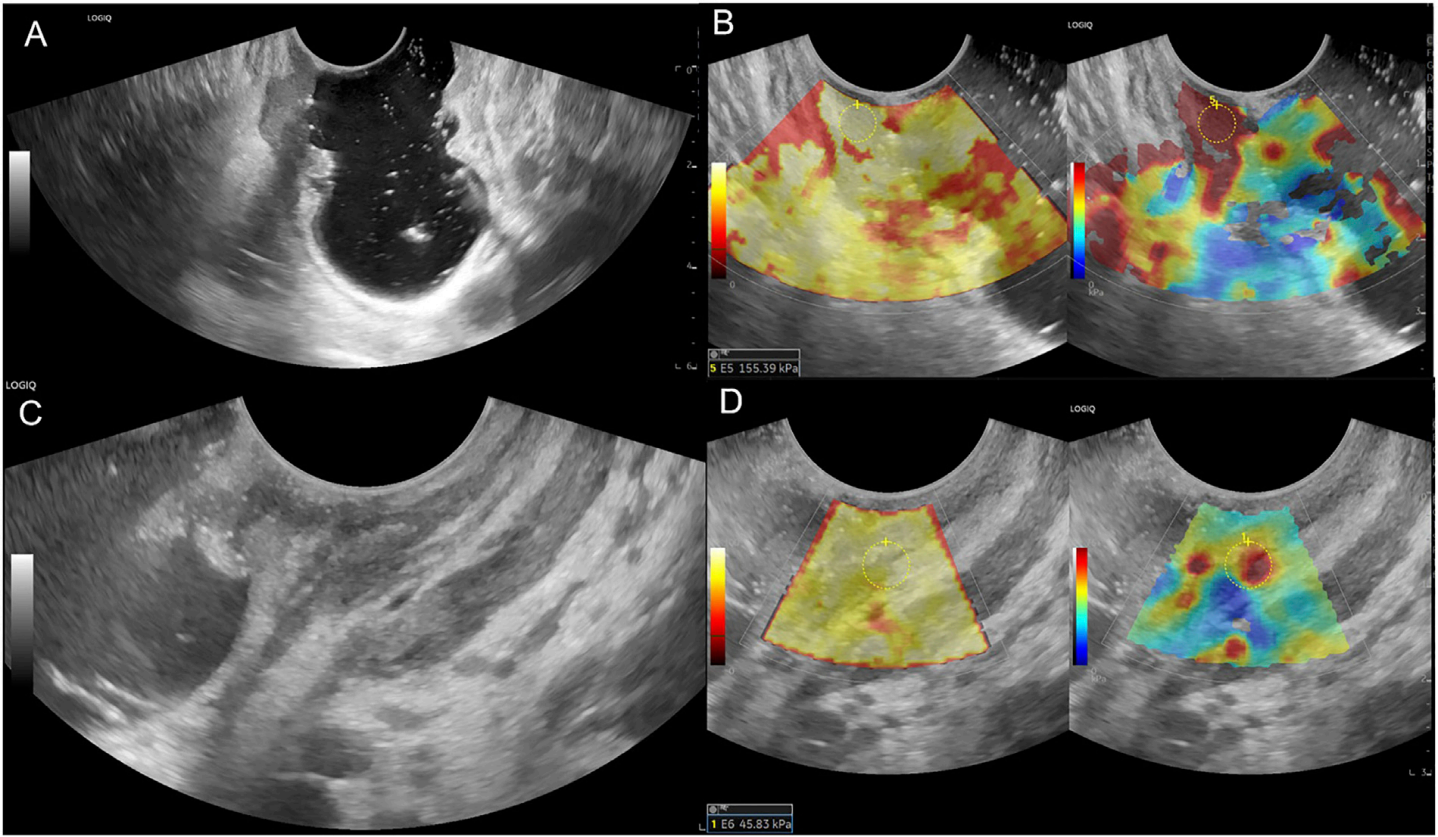
Female patient, 69 years old, **(A)** Lower rectum circumferential ulcerative lesion, gray-scale shown disruption of the rectal mucosa, submucosa, muscularis and serosa, ERUS staged uT3; **(B)** SWE showed the lesion is tough, pre-nCRT Emean = 155.39 kPa, pre-nCRT EC = 149.19 kPa; **(C)** after nCRT, rectal mucosa, submucosa and muscularis were thickened in the front wall, the intestinal wall is unclearly layered, ERUS staged uT2; **(D)** SWE showed the lesion area turn to be soft, post-nCRT Emean = 45.83kPa, post-nCRT EC = 34.33kPa, ED = 109.56 kPa, ECD = 114.86kPa, EDR = 70.51%, ECDR = 76.99%, pathological result is ypT0N0.

**FIGURE 4:**
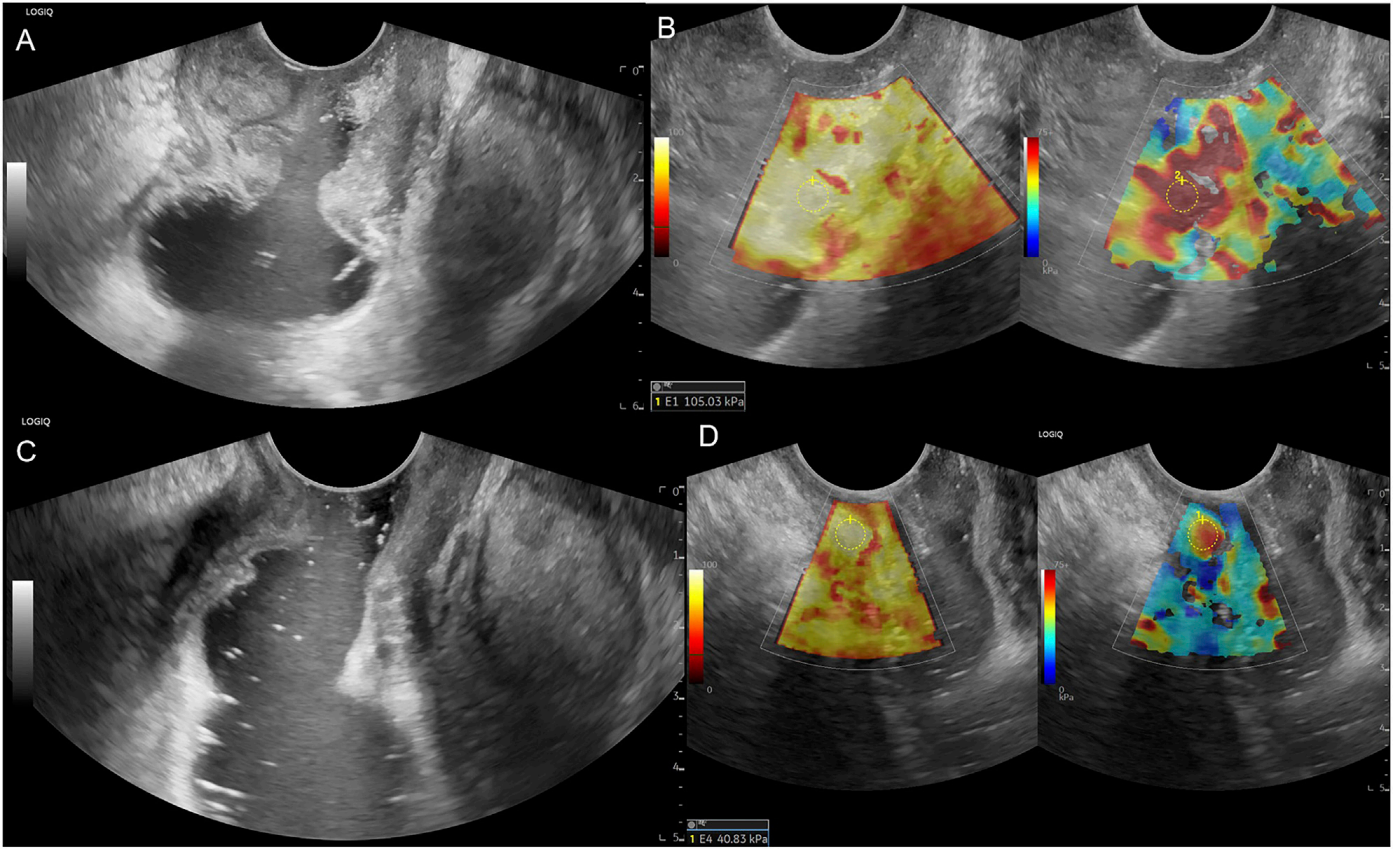
Male patient, 54 years old, **(A)** Low rectum circumferential protuberant lesion, gray-scale shown disruption of the rectal mucosa, submucosa, muscularis, serosa and periintestinal fat tissue, ERUS staged uT3; **(B)** SWE showed the lesion is almost in red colour, pre-nCRT Emean = 105.03 kPa, pre-nCRT EC = 101.03kPa; **(C)** after nCRT, the wall of low rectum was circumferential thickened, the intestinal mucosa, submucosa, muscularis, serosa seems unclearly layered, ERUS staged uT3; **(D)** SWE showed the lesion area turn soft, post-nCRT Emean = 40.83kPa, post-nCRT EC = 29.83kPa, ED = 64.20 kPa, ECD = 71.20 kPa, EDR = 61.13%, ECDR = 70.47%, pathological result is ypT0N0.

Table 1 presents the results of the uT stage diagnosed using ERUS after nCRT compared with the postoperative pathological ypT stage. Eighteen patients (18/31, 58.1%) were correctly staged using ERUS, whereas six cases were overstaged and seven were understaged. The accuracy of conventional ERUS in predicting the ypT stage after nCRT was 58.1% (18/31). The accuracy in predicting the ypT0 stage was 64.3%; of the 14 ypT0 stage patients, nine were correctly diagnosed (Figure 2). However, among the five misdiagnosed patients, four were misdiagnosed as the uT1-T2 stage (Figure 3) and one as the uT3 stage (Figure 4).

**TABLE 1:** Thirty-one patients’ ultrasound T (uT) stage after neoadjuvant chemoradiotherapy and pathological T (ypT) stage after surgery.

Pathological T stage	Ultrasound T stage				
	uT0	uT1	uT2	uT3	uT4
ypT0	9	2	2	1	0
ypT1	0	1	0	0	0
ypT2	1	2	3	1	0
ypT3	0	2	1	5	0
ypT4	0	1	0	0	0

uT:ultrasound T stage, ypT:yield pathological T stage.

### SWE evaluates T stage

The Emean and EC values decreased in 29 patients and increased slightly in two patients after nCRT. The Emean values of lesions before and after nCRT were 126.43 ± 21.78 kPa and 71.21 ± 30.13 kPa, respectively, and the difference was statistically significant (t = 10.51, *p* < 0.001). The EC values of lesions before and after nCRT were 117.57 ± 22.76 kPa and 61.64 ± 30.35 kPa, respectively (t = 10.43, *p* < 0.001). The Emean values of the normal intestinal wall were 8.85 ± 3.13 kPa and 9.58 ± 2.12 kPa, respectively; the difference was not statistically significant (t = −1.19, *p* = 0.24).

Only one patient each had ypT1 and ypT4 stage, respectively. Therefore, for analysis, ypT1 and ypT2 with tumors confined to the intestinal wall were grouped into the ypT1-2 group, and ypT3 and ypT4 with tumors invading the outside of the intestinal wall were grouped into the ypT3-4 group. Table 2 lists the Emean and EC values in each group after nCRT. After nCRT, the Emean and EC values increased with increasing tumor stage, and the Emean and EC values of lesions at different pathological stages were significantly different (*p* = 0.006). There were statistically significant differences between the ypT0 and ypT1-2 groups (*p* = 0.031) and between the ypT0 and ypT3-4 groups (*p* < 0.001), whereas the ypT1-2 and ypT3-4 groups showed no significant difference (*p* = 0.686).

**TABLE 2:** post-nCRT Emean values and post-nCRT EC values of rectal tumor lesions in different T staging after nCRT.

	Pathological T staging after surgery		
	ypT0	ypT1-2	ypT3-4
post-nCRT Emean (kPa)	48.14 ± 19.11	80.13 ± 21.80	99.17 ± 22.49
post-nCRT EC (kPa)	38.40 ± 18.40	70.04 ± 21.74	90.31 ± 23.45

pre-nCRT: before neoadjuvant chemoradiotherapy, post-nCRT: after neoadjuvant chemoradiotherapy, EC (kPa): Emean corrected value.

**FIGURE 5:**
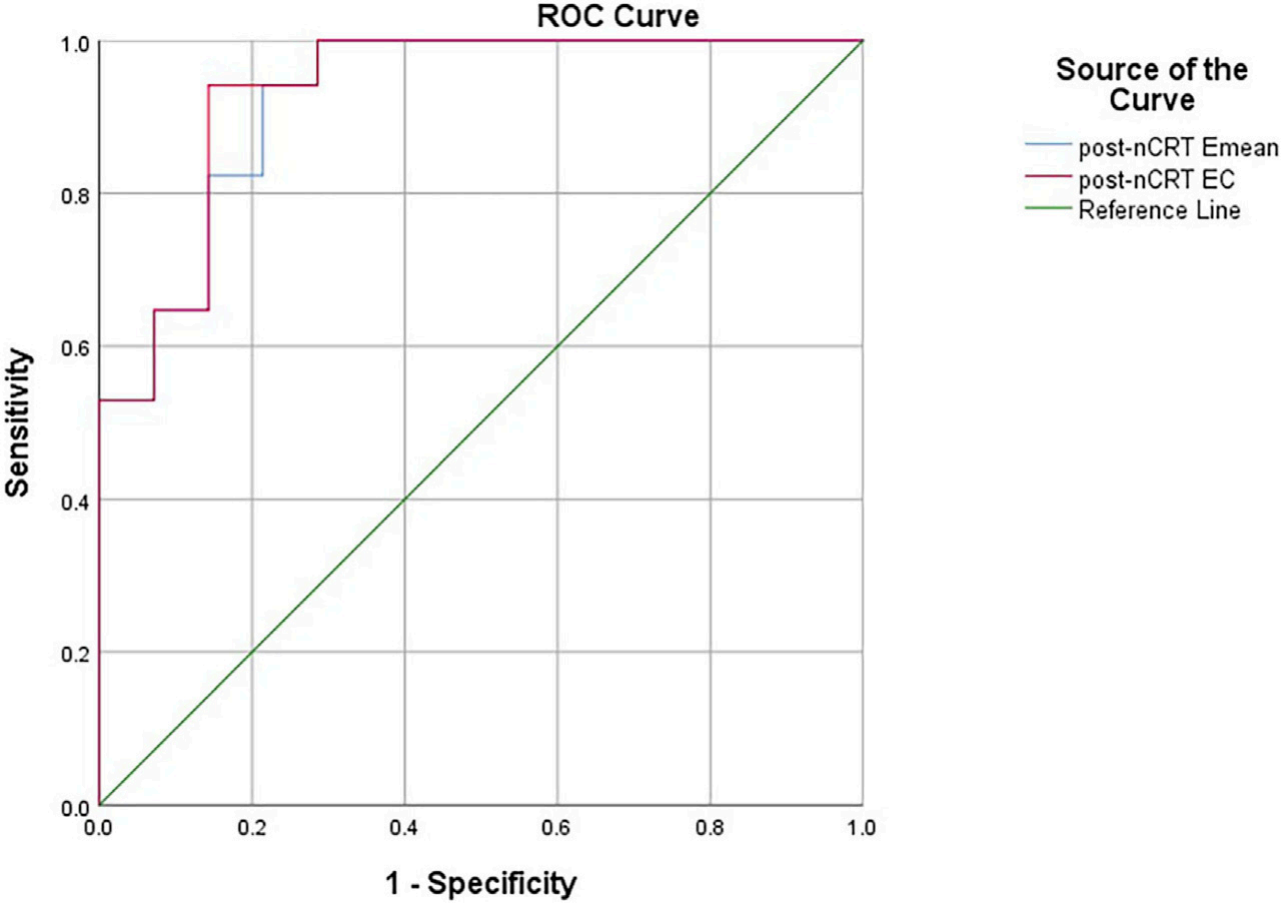
The receiver operating characteristic curves constructed with the lesions’ post-nCRT Emean and post-nCRT EC values for diagnosing the ypT0 stage. pre-nCRT: before neoadjuvant chemoradiotherapy, post-nCRT: after neoadjuvant chemoradiotherapy, EC (kPa): Emean corrected value.

The ROC curves constructed with the lesions’ post-nCRT Emean and post-nCRT EC values showed that the best diagnostic cut-off values for diagnosing the ypT0 stage were 64.40 kPa and 55.45 kPa, respectively. The area under the curves (AUC) were 0.924 (95% confidence interval [CI]: 0.832–1) and 0.933 (95% CI: 0.844–1), respectively (Figure 5). Emean <64.40 kPa was used for stage uT after nCRT, and the sensitivity, specificity, and accuracy of the ypT0 stage were 94.12%, 78.60%, and 92.86% (13/14), respectively. EC < 55.45 kPa was used for stage uT after nCRT, and the sensitivity, specificity, and accuracy of the ypT0 stage were 94.12%, 85.70%, and 92.86% (13/14), respectively.

**FIGURE 6:**
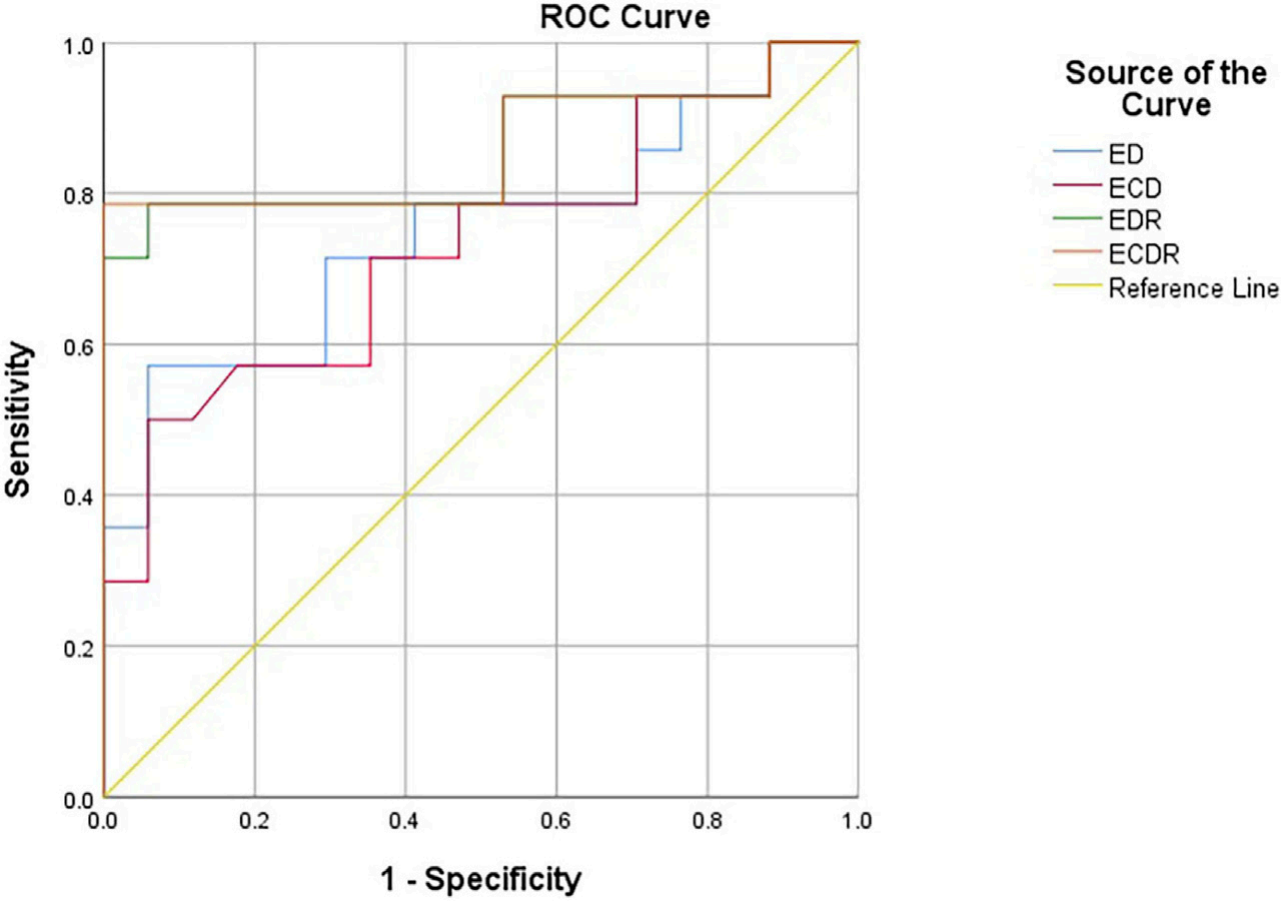
The receiver operating characteristic curves constructed with ED, ECD, EDR and ECDR for diagnosing the ypT0 stage. ED (kPa): Emean difference, ECD (kPa): Emean corrected differencede, EDR (%): Emean descendding rate, ECDR (%):Emean corrected descendding rate.

The ROC curves constructed with the lesions’ ED, ECD, EDR, and ECDR showed that the best diagnostic cut-off values for diagnosing the ypT0 stage were 72.55 kPa, 73.75 kPa, 50.15%, and 55.93%, respectively (Figure 6). Table 3 summarizes the sensitivity, specificity, and AUC for diagnosing the ypT0 stage. ED > 72.55 kPa was used for stage ypT0 after nCRT, and the sensitivity, specificity, and accuracy were 57.14%, 94.11%, and 85.71% (12/14), respectively. ECD >73.75 kPa was used for stage ypT0 after nCRT, and the sensitivity, specificity, and accuracy were 50.00%, 94.11%, and 85.71%, respectively. EDR >50.15% was used for stage ypT0 after nCRT, and the sensitivity, specificity, and accuracy were 78.57%, 94.11%, and 92.86% (13/14), respectively. ECDR >55.93% was used for stage ypT0 after nCRT, and the sensitivity, specificity, and accuracy were 78.57%, 100%, and 92.86% (13/14), respectively.

**TABLE 3:** Results of ROC curves of ED, ECD, EDR and ECDR.

SWE	Cut-off value (kPa)	Sensitivity (%)	Specificity (%)	AUC
ED (kPa)	72.55	57.14	94.11	0.748(95% CI:0.565–0.931)
ECD (kPa)	73.75	50.00	94.11	0.729(95% CI:0.544–0.914)
EDR(%)	50.15	78.57	94.11	0.857(95% CI:0.705–1)
ECDR (%)	55.93	78.57	100	0.861(95% CI:0.709–1)

ED (kPa): Emean difference, ECD (kPa): Emean corrected differencede, EDR (%): Emean descendding rate, ECDR (%): Emean corrected descendding rate.

### Analyzing patients with cCR

We also evaluated the patients with a clinical diagnosis of cCR and showed that the average post-nCRT Emean and EC values of the lesions were 40.57 ± 20.76 kPa and 31.89 ± 20.39 kPa, respectively. Seven patients had the coincident Emean and EC values with the cut-off values for diagnosing the ypT0 stage, and the accuracy was 77.78% (7/9), respectively. The ED, ECD, EDR, and ECDR values were 95.67 ± 21.98 kPa, 95.04 ± 22.06 kPa, 70.53% ± 13.28%, and 75.25% ± 14.42%, respectively. Eight patients had the coincident ED, ECD, EDR, and ECDR values with the cut-off values for diagnosing the ypT0 stage, and the diagnostic accuracy was all 88.89% (8/9).

## Discussion

The recent gradual individualization and precision of treatment regimens and the development and application of new drugs, such as immunotherapy (programmed death-1/programmed death-ligand 1 monoclonal antibody), have improved rectal cancer treatment. Approximately 13.5%–40% of patients with rectal cancer who initially cannot undergo surgery or achieve R0 resection (Fernández-Martos et al., 2010; Nilsson et al., 2013; Garcia-Aguilar et al., 2015; Perez et al., 2017; Zhang et al., 2020) can achieve pCR after nCRT. This study was conducted simultaneously with a clinical randomized controlled trial at our hospital’s Radiation Oncology Department. Among them, 45.16% of patients achieved pCR after nCRT, a higher proportion compared with previous studies. This may be associated with the clinical application of new drugs and our hospital’s precise delineation of radiation therapy target volumes.

The consensus guidelines (Chinese Watch & Wait Database Research Cooperation Group et al., 2020; Temmink et al., 2023) suggest no significant difference in survival rates between patients with cCR after nCRT who received the W&W strategy and those with pCR. Compared with radical surgery, the W&W strategy can significantly improve patients’ quality of life without compromising treatment efficacy (Renehan et al., 2016). Currently, there are varying standards for determining cCR, including endoscopy, digital rectal examination, transrectal ultrasonography, rectal MRI T2 weighted image/DWI sequences, and serum carcinoembryonic antigen (CEA) (Habr-Gama et al., 2013; Glynne-Jones et al., 2018; Temmink et al., 2023). However, currently established cCR criteria cannot accurately determine pCR. There was only a partial overlap between the cCR and pCR groups. A systematic review reported that among patients diagnosed with cCR preoperatively, the proportion of those with pCR postoperatively was only 30% (Glynne-Jones et al., 2008), indicating a high rate of misdiagnosis. Among the 14 patients with the ypT0 stage in this study, colonoscopy results suggested residual tumor in seven patients, and high signals on the DWI sequence of MRI indicated residual tumor in seven patients. In misdiagnosed patients, radical surgery may cause overtreatment and affect their long-term quality of life. Therefore, improving the accuracy of the yielded pCR prediction has become crucial.

SWE has recently become a popular imaging modality. It has been widely used for the differential diagnosis of benign and malignant breast lesions, thyroid nodules, prostate lesions, musculoskeletal diseases, and liver cirrhosis (Zhou et al., 2014; Taljanovic et al., 2017; Anbarasan et al., 2021; Gao et al., 2022; Luo et al., 2022). However, there are relatively few studies on its application to T-staging diagnosis of rectal cancer. SWE does not require manual compression by an operator compared with traditional strain elastography. It measures the Young’s modulus value (Emean), representing the stiffness. In this study, we performed ERUS by filling the rectum with sterile coupling gel (Wang et al., 2012). The probe did not need to press the bowel wall, and a clear view of the rectum and tumor was obtained without compression. This approach minimizes the operator’s influence and makes the measurements more objective. Notably, most studies on SWE for tumor diagnosis have used Emean or Emax values as the diagnostic criteria (Zhou et al., 2014; Taljanovic et al., 2017; Anbarasan et al., 2021; Gao et al., 2022; Luo et al., 2022). There are differences in the selection of diagnostic indicators for different tumors. Emax has shown a better diagnostic performance for breast cancer diagnosis (Zhou et al., 2014). Currently, there is no consensus on the choice of stiffness measurement for patients with rectal cancer. A previous study using the AixPlorer ultrasound instrument from Supersonic Imaging demonstrated that Emax has good diagnostic value for assessing the T-staging of rectal cancer after nCRT (Cui et al., 2020). Another study suggested that Emean had a better diagnostic value than did Emax (Loft et al., 2022b). Therefore, in this study, we selected the Emean as the research indicator to further validate the diagnostic value of different E values. The GE LOGIQ E11 diagnostic apparatus used in this study is popular in hospitals in China, making it easier to perform ERUS and SWE in patients and promoting multicenter cooperation in the future.

After nCRT, tumor lesions undergo pathological changes, such as tumor cell necrosis, infiltration of lymphocytes and megakaryocytes, and proliferation of connective tissue around the lesion (Dworak et al., 1997). These changes alter the tumor’s physical properties, including its stiffness. In this study, 29 patients had decreased post-nCRT Emean and EC values, whereas two showed a slight increase. Consistent with previous studies (Cui et al., 2020; Tang et al., 2024), the overall post-nCRT Emean values were significantly lower compared with those measured before treatment. Regarding the two patients with a slight increase in post-nCRT Emean values, postoperative pathology showed no decrease in the ypT stage. Therefore, the increase in Emean value may be associated with tumor progression, increased number of tumor cells, or fibrosis within the lesion. In this study, the Emean and EC values increased with the tumor T stage after nCRT. Specifically, there were significant differences in these values between the ypT0 and ypT1-2 groups and between the ypT0 and ypT3-4 groups. However, no significant differences were observed between the ypT1-2 and ypT3-4 groups. This could be because the sample size was relatively small, and the statistical power was insufficient, resulting in no statistical difference.

In this study, the accuracy of conventional ERUS for T staging after nCRT was only 58.1%, similar to previous studies (Dickman et al., 2013; Zhao et al., 2014; Cong et al., 2017; Chen et al., 2019; Cong et al., 2019; Tang et al., 2024). Among them, six patients, mainly patients with ypT0 (5/6), were overstaged. This may be because after radiotherapy and chemotherapy, fibrotic tissue replaces the tumor, and grayscale ultrasound shows poor definition and irregular borders of the bowel wall layers (Figures 3C, 4C), which cannot distinguish between fibrotic tissue and tumor lesions, leading to its misdiagnosis as a residual tumor. Seven cases were understaged, possibly due to the presence of only a very small amount of residual tumor infiltrating the muscularis propria or mucosal layer after radiotherapy and chemotherapy, which was difficult to detect using grayscale ultrasound. This study’s accuracy of ERUS in diagnosing the ypT0 stage was 64.3%. Among the 14 patients who achieved a complete response after nCRT, only nine were accurately diagnosed using ERUS, whereas five were misdiagnosed. Among them, four were misdiagnosed as the uT1-T2 stage and one as the uT3 stage. The diagnostic accuracy was low, consistent with previous studies (Fan et al., 2019; Cong et al., 2021). The Emean and EC values represent the lesion’s absolute stiffness. Using a cut-off Emean value of <64.4 kPa after nCRT to diagnose the ypT0 stage, we had a sensitivity, specificity, and accuracy of 94.12%, 78.60%, and 92.86% (13/14), respectively.

Similarly, using a cut-off value of EC < 55.45 kPa after nCRT to diagnose ypT0, we had a sensitivity, specificity, and accuracy of 94.12%, 85.70%, and 92.86% (13/14), respectively. The diagnostic accuracy of EURS combined with Emean and EC values was significantly improved compared with that of conventional ERUS. We measured the Emean values of a normal bowel wall before and after nCRT in the same patient to exclude individual differences in bowel wall stiffness, and found that the post-nCRT EC value, which excluded the interference of bowel wall stiffness, had a higher sensitivity, specificity, and AUC in the ROC curve, indicating better diagnostic performance. We derived relative stiffness parameters, including the lesion ED, ECD, EDR, and ECDR, by comparing the stiffness of lesions before and after nCRT. The AUC of the ROC curves were 0.748, 0.729, 0.857, and 0.861, respectively. These relative stiffness parameters had slightly lower diagnostic performances than did the absolute stiffness values. However, using the cut-off values of these SWE parameters to re-stage uT significantly improved the accuracy of diagnosing the ypT0 stage to 85.71%–92.86%. As shown in Figures 3C, 4C, these two patients were in the ypT0N0 stage postoperatively, ERUS showed thickening and unclear layers of the lesions on grayscale after nCRT, and they were diagnosed with uT2 and uT3 stages, respectively. As shown in Figures 3D, 4D, the SWE examination showed that the lesions were soft. After Emean measurement and calculation, SWE parameters were consistent with the diagnostic cut-off value of the ypT0 stage, indicating that ERUS combined with SWE could achieve an accurate re-staging diagnosis. The SWE parameters in our study could improve the accuracy of predicting ypT0 stage of rectal cancer after neoadjuvant therapy, help clinicians judge the cCR status in patients after nCRT, and provide strong support for the W&W strategy. In addition, SWE is easy to operate; provides objective data; and easily promotes and applies to different hospitals and equipment, which can be directly applied to clinical work and benefit patients.

Nine patients were clinically diagnosed with cCR and did not undergo surgery. Statistical analysis showed that the SWE parameters after nCRT in these patients were 77.78%–88.89%, consistent with our study’s diagnostic cut-off values. No local recurrences or distant metastases were observed.

This study has some limitations. First, it was a single-center study, and there may have been bias in case selection. Second, the sample sizes for this study’s different T stages were unbalanced. Notably, some patients with uT1 refused surgical treatment due to the inability to preserve the anus. However, patients with uT4 were often unsuitable for TME surgery owing to the lack of radical resection indications. Therefore, only one patient each had ypT1 and ypT4 stage, respectively. Consequently, it was impossible to analyze the diagnostic efficacy of SWE in each T stage. Third, this was a prospective study. However, the sample size was relatively small, and some patients refused surgery or could not undergo radical resection. Only 31 of 60 patients received surgical treatment, resulting in a small number of patients with pathological outcomes. In the future, with enough long duration of time and large number of patients, the diagnostic effect of SWE can be evaluated more comprehensively to further validate the conclusions in this study. The application of SWE in patients with rectal cancer after nCRT is not only limited to the prediction of ypT0, but also to the diagnostic cut-off values of other T stages and monitoring for local recurrence in patients with cCR.

In conclusion, the SWE parameters in this study improved the diagnostic accuracy of ERUS for predicting ypT0 stage after nCRT; the EC value showed the best diagnostic performance. These findings provide more evidence for clinically and accurately diagnosing patients with cCR. The results can be used as a pilot study. In future studies, we will expand the sample size, promote multicenter cooperation, and further validate the accuracy of the relevant parameters.

## Data Availability

The original contributions presented in the study are included in the article/Supplementary material, further inquiries can be directed to the corresponding author.
